# Health State Estimation of On-Board Lithium-Ion Batteries Based on GMM-BID Model

**DOI:** 10.3390/s22249637

**Published:** 2022-12-08

**Authors:** Shirui Feng, Anchen Wang, Jing Cai, Hongfu Zuo, Ying Zhang

**Affiliations:** 1 College of Automobile and Traffic Engineering, Nanjing Forestry University, Nanjing 210037, China; 2 School of Civil Aviation, Nanjing University of Aeronautics and Astronautics, Nanjing 210016, China

**Keywords:** lithium-ion battery, health state estimation, multi-source information fusion, gaussian mixture model

## Abstract

As a single feature parameter cannot comprehensively evaluate the health status of a battery, a multi-source information fusion method based on the Gaussian mixture model and Bayesian inference distance is proposed for the health assessment of vehicle batteries. The missing and abnormal data from real-life vehicle operations are preprocessed to extract the sensitive characteristic parameters which determine the battery performance. The normal state Gaussian mixture model is established using the fault-free state data, whereas the Bayesian inference distance is constructed as an index to quantitatively evaluate the battery performance state. In order to solve the problem that abnormal data may exist in the measured data and introduce errors into evaluation results, the determination rules of abnormal data are formulated. The verification of real-life vehicle operation data reveals that the proposed method can accurately evaluate the onboard battery state and reduce safety hazards of electric vehicles during the normal operation process.

## 1. Introduction

At present, lithium-ion batteries (LIBs) are widely used in energy vehicles by major automobile enterprises because of their high energy density, high average output voltage, superior cyclic performance, and rapid charge–discharge. However, the structural mechanism of lithium-ion batteries is quite complex and there are significant safety risks in the process of using onboard batteries. A risk of fire is associated with the occurrence of internal faults. Additionally, the spontaneous combustion of the battery pack directly leads to the fire and spontaneous combustion of the electric vehicle. As a key component of pure electric vehicles, battery degradation or failure affects the normal operation of the vehicle and raises serious safety concerns. Therefore, it is of great significance to evaluate the health status of onboard batteries. 

Onboard battery condition assessment methods are developing rapidly, mainly from the perspective of a single battery or battery pack [1,2,3]. Luciani et al. [4] presented the design and hardware-in-the-loop (HIL) experimental validation of a data-driven estimation method for the state of charge (SOC) of LIBs used in hybrid electric vehicles (HEVs). The resulting estimation algorithm can estimate the battery SOC in real time with 2% accuracy during real-time hardware testing. Chen et al. [5] have proposed an online battery health state assessment method based on battery model parameters, utilizing a genetic algorithm to estimate the parameters of a battery model, finding diffusion-controlled capacitance, and deriving an equation for SOH estimation. Widodo et al. [6] utilized the sample entropy of discharge voltage as a characteristic parameter to characterize the health status of battery performance and predicted the remaining battery life based on the sample entropy. Krupp et al. [7] proposed a method to identify non-uniform aging states on the capacitance vs. capacity curve of a battery pack and evaluate the status of single cells and serial- or parallel-connected battery packs. Moreover, the validity of the proposed method is assessed by experimental characterization. Toughzaoui et al. [8] combined the long-short-term memory (LSTM) network to estimate the health of LIBs. Weng et al. [9] applied incremental capacity analysis to assess the health status of LIBs by tracking the peak of incremental capacity analysis curve and experimentally verified the method’s applicability to both single cells and battery packs. Wang et al. [10] proposed a new algorithm for differential voltage curve acquisition based on the central least square method for battery health assessment, extracting the change in the position of spike at the end of curve as a characteristic parameter to characterize the health status of a battery pack, as well as experimentally verifying the effectiveness of the proposed method. These studies on cell and battery packs are limited in application due to the difficulty of obtaining accurate cell data.

With the development of machine learning algorithms, many data-driven methods are gradually being applied to battery status assessment [11,12,13], such as support vector machines, correlation vector machines, neural networks, etc. [14,15,16] Jiechun Liang et al. [17] listed the advantages of artificial intelligence and the application steps of artificial intelligence in their research, including data availability, selection of training algorithms, and interpretation of results. Qin Deng et al. [18] proposed a new method combining extreme feature engineering and automatic machine learning. A large number of new descriptors are constructed by extreme feature engineering and the key subsets are obtained by a sequential forward selection algorithm. Using linear regression to determine the best descriptor provides a new way for research. Wei et al. [19] proposed an assessment method for the state of health of echelon utilization batteries based on deep neural network learning with error correction and the results revealed that the average absolute errors of the state of health prediction for echelon utilization batteries are less than 0.8%. Then, the prediction model is modified by Markov chain error, which provides a theoretical basis for the safe and stable operation of batteries. Bi et al. [20] proposed a battery SOH evaluation model based on genetic resampling particle filtering to solve the problem of multi-source noise in the simplified equivalent circuit model of a LIB pack, which leads to the non-Gaussian nature of the system and verifies the superiority of the proposed method. Guha et al. [21] provided a structured approach to monitor the SOH of a battery, built an empirical model of battery capacity degradation and internal resistance growth for end-of-life prediction at various stages using a particle filtering framework, and combined both models to obtain a novel degradation model for remaining battery life estimation. Xu et al. [22] investigated the effect of relaxation on the degradation pattern of Li-ion batteries and proposed a new SOH estimation method based on the Wiener process, which divided the life cycle of LIBs into three processes and established the corresponding degradation models, effectively improving the assessment accuracy. Most of them use a single feature to evaluate the battery condition, and the accuracy of the evaluation needs to be further improved. 

Based on the operating data of battery packs, the current paper adopts multi-source information fusion technology to extract features from various types of data and combines multiple parameters into quantitative indicators, characterizing and evaluating the health status of a battery pack. The proposed method provides a novel research direction for the condition assessment of a battery pack, improves the accuracy of onboard battery condition assessment, reduces maintenance cost caused by battery abnormalities, and renders important research significance for comprehensively improving the technology level, seaworthiness, and safety of electric vehicles.

## 2. Data Pre-Processing and Feature Parameter Selection

There are certain problems due to equipment issues or changes in the external environment, such as missing data and abnormal data. Therefore, it is necessary to effectively process the actual driving data of electric vehicles to improve the credibility of research results. In addition, a single feature parameter cannot comprehensively reflect the overall state of the vehicle battery. Hence, it is necessary to analyze and select the feature parameters in the vehicle operation data to reflect the real state of a battery comprehensively and completely.

### 2.1. Data Introduction and Preprocessing

The actual driving data of electric vehicles used are obtained from the usual drive of 62 vehicles of a certain type of pure electric vehicle in 117 days from 26 August 2020 to 31 December 2020. The sampling period is 10 s. Sampling data types include real vehicle driving data acquisition time, vehicle status, operation mode, speed and mileage accumulation, SOC, gear, insulation resistance, accelerator pedal stroke, number of motors, motor speed, motor, motor-controlled input voltage, temperature controller, highest level alarm, battery voltage, subsystem number, total battery voltage, total current of the battery pack, number of battery strings, voltage of each battery string, battery string temperature, number of probes, and temperature of each probe. The data format is shown in Table 1. The preprocessing of data is carried out to complete missing data and eliminate abnormal data.

#### 2.1.1. Missing Data Processing

When the data acquisition device of the vehicle terminal transmits data to the data center, there may be data loss or data failure, resulting in missing data points. Herein, the weighted moving average is used to fill in missing data. When the weighted moving average method is used to interpolate missing data, each missing value is supplemented by the weighted average of *k* observations on both sides, which is called the window size. Let $\left\{Y_{t},t=1,\cdots ,T\right\}$ be the target time series and then, the sliding weighted average is defined as follows [23]:

(1) $${\hat{Y}}_{t+1}=\sum_{i=k}^{k}\omega_{i}Y_{t+1+i}$$

where $\omega_{-k},\omega_{-k+1},\cdots ,\omega_{k}$ refers to the weight of each moment point, and the weight value is cumulatively equal to 1. If there is a situation where the window is not available due to missing values, the k value is incremented. The weight at each moment point can be classified as equal (simple) weights, linear weights, and exponential weights. Based on the experience, the window length is considered as 2 in this paper. Considering the close relationship between the data before and after and the missing data, the weight value of the data closer to the missing position is larger and the weight value of the data farther away from the missing position is smaller. For example, if the length of the window is 3, the data weight of the four points before and after will be changed into 1/14, 1/7, 2/7, 2/7, 1/7, 1/14 in chronological order, which has little effect on the improvement of the accuracy of identifying missing data. Therefore, based on engineering experience, the window length is considered as 2. 

Taking certain data as an example, 10 data types, including vehicle speed, cumulative mileage, SOC, insulation resistance, motor speed, motor temperature, input voltage of motor controller, DC bus current of motor controller, total voltage of the battery pack, and total current of battery pack, were taken to perform interpolation of missing data of weighted sliding average. Table 2 presents the data of two time points before and after missing data, i.e., the window set. According to the principle of weighted sliding average, the data weights of four points before and after being set are 1/3, 1/6, 1/3, and 1/6, respectively, in chronological order. The obtained results are shown in the third row of Table 2.

#### 2.1.2. Handling Abnormal Data

**Figure 1:**
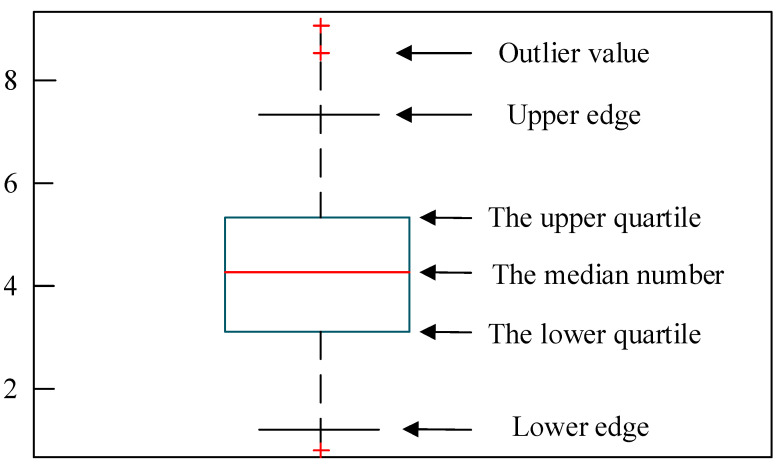
Box-type plot.

Due to some equipment issues or changes in the external environment, there are some outliers during the process of data acquisition of the vehicle terminal. In order to improve the precision of the study, the box graph method [24] was used to deal with the outliers in the data. The structure of the box diagram is shown in Figure 1. The process of eliminating outliers in the box diagram is as follows:

(1) Input data: $x_{i},i=1,2,\ldots ,n$
(2) The values of lower quartile ($Q_{1}$), upper quartile ($Q_{3}$), and IQR are determined as follows:
  (2) $$IQR=Q_{3}-Q_{1}$$
(3) Sample: The parameter k was set to calculate the value of $B_{1}\left(k\right),B_{2}\left(k\right)$, as follows:
  (3) $$B_{1}\left(k\right)=Q_{3}+k\cdot IQR$$
  (4) $$B_{2}\left(k\right)=Q_{1}-k\cdot IQR$$
(4) $x_{i}$ is defined as a mild outlier when $x_{i}\in \left[Q_{3},B_{1}\left(1.5\right)\right]$ or $x_{i}\in \left[B_{2}\left(1.5\right),Q_{1}\right]$
(5) $x_{i}$ is defined as an extreme outlier when $x_{i}\in \left[B_{1}\left(1.5\right),B_{1}\left(3\right)\right]$ or $x_{i}\in \left[B_{2}\left(3\right),B_{2}\left(1.5\right)\right]$
(6) The data corresponding to outliers were eliminated.

**Figure 2:**
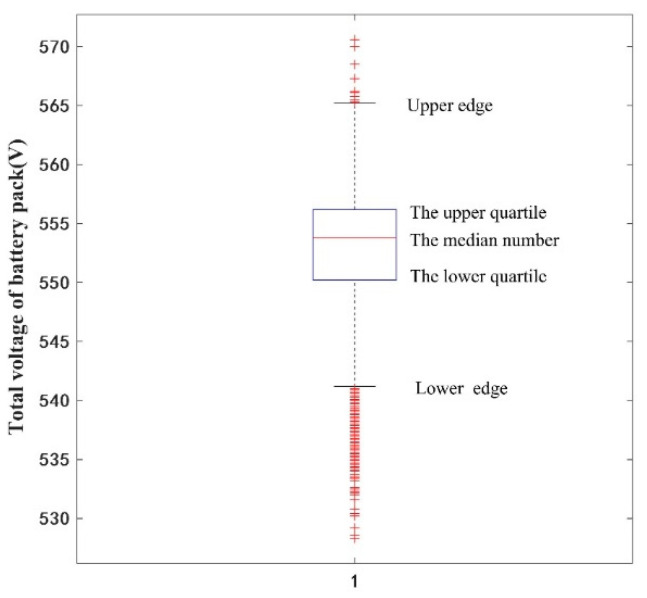
A box-type plot of the total voltage of a battery pack.

Figure 2 presents the box-type diagram of the total voltage of a battery pack. It can be observed that the total voltage of the whole battery pack is distributed in the range of 528.3 to 570.6 V. The lower edge value of the total voltage of a battery pack is 541.2 V, upper quartile is 556.2 V, median is 553.8 V, lower quartile is 550.2 V, and upper edge value is 565.2 V. According to the theory of the box diagram, outliers beyond the upper and lower edge values should be eliminated, i.e., the data of the total battery voltage between 528.3 V and 541.2 V, and 565.2 V and 570.6 V, should be eliminated.

The actual operation data of pure electric vehicles were analyzed and processed from 26 August 2020 to 31 December 2020, with a total of 140,393 running state data. Firstly, the data types obtained are analyzed and the data types without analytical value are removed to reduce the dimensions of the data. Then, the missing data in the dataset were analyzed. The long-term missing data were deleted, and the short-term missing data were interpolated by weighted sliding averages. A total of 4467 missing data were deleted and 39 missing data were completed. Finally, abnormal data are detected and deleted by the box-type graph method. At this point, all data preprocessing work has been completed and the amount of data was 59,283.

### 2.2. Feature Parameter Selection

**Figure 3:**
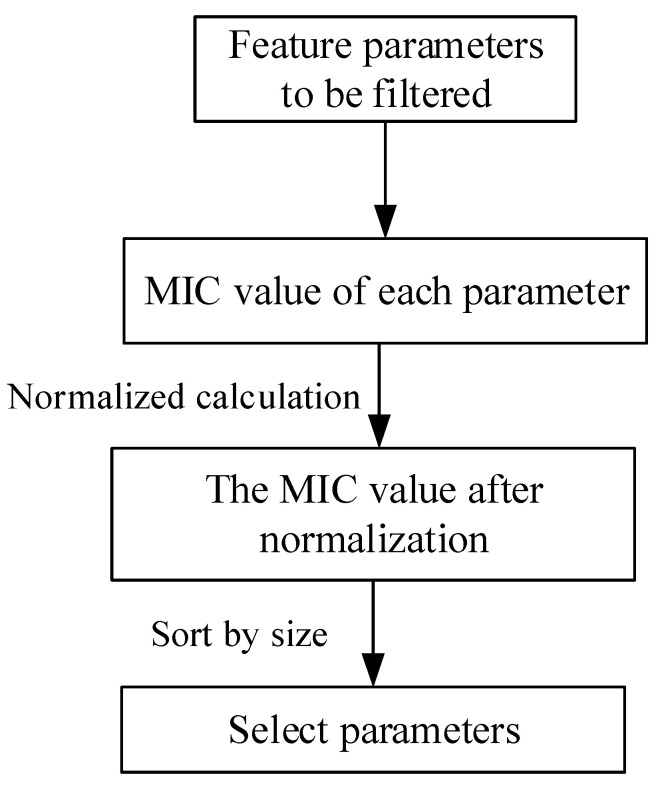
Feature parameter selection process.

When condition assessment cannot fully reflect the overall vehicle battery state, and considering the single characteristic parameters, the choice of characteristic parameters of the vehicle running in the data that reflect the real status of a battery should be more comprehensive. Herein, the feature selection method of maximum information coefficient is used to select the features that can characterize the state of onboard batteries. The selection process of feature parameters is shown in Figure 3. The feature selection process based on the maximum information coefficient (MIC) is as follows:

- Select the feature parameter that can best characterize the health status of the onboard battery and select other features that can assist in jointly characterizing the health status of the battery.
- Calculate the maximum information coefficient between the feature parameter that can best characterize the health state of the onboard battery and other auxiliary feature parameters.
- Calculate the mean value of all maximum information coefficients, retain the features with maximum information coefficients greater than the mean value, and eliminate the features with maximum information coefficients less than the mean value.
- Normalize the MIC values of all obtained features to the interval [0, 1] and rank all MIC values in descending order to obtain the ranking of features and further validate the screened features.

The maximum information coefficient is calculated as follows:

There are n points in the data set $D=\left\{\left(a_{1},b_{1}\right),\ldots ,\left(a_{n},b_{n}\right)\right\}$ and a two-dimensional scatter plot of the data set is drawn with the horizontal and vertical coordinates. The grid X×Y is denoted by $G\left(X,Y\right)$ and $p_{0},\ldots ,p_{x}$ indicate the segmentation points on the horizontal axis (where $p_{0}=a_{1},p_{x}=a_{n}$). Similarly, $q_{0},\ldots ,q_{y}$ represent the segmentation points on the vertical axis, where $q_{0}=b_{1},q_{y}=b_{n}$. The values of $p_{1},\ldots ,p_{x-1}$ and $q_{1},\ldots ,q_{y-1}$ are varied to obtain different segmentation methods in the X×Y grid. Let $\max I\left\{x,y\right\}$ denote the maximum mutual information value that can be obtained by different grid partitioning methods in the grid $G\left(X,Y\right)$, the value of the feature matrix calculated by this partitioning method can be given as:

(5) $$M_{x,y}=\frac{\max I\left\{x,y\right\}}{\log\min\left(x,y\right)}$$

Then, the maximum correlation coefficient (MIC) can be given as:

(6) $$\mathrm{MIC}=\max\left\{M_{x,y}\right\}$$

Since there are multiple grid division methods, a parameter $B\left(n\right)$ is introduced to improve the computational efficiency, which is an upper limit of the grid size and is used to limit the number of grid division methods. If the $B\left(n\right)$ value is too low, it will be challenging to find the optimal grid division, resulting in imprecise MIC. If $B\left(n\right)$ value is too high, the grid division becomes too dense and the problem of one point per grid occurs. The value of $B\left(n\right)$ in Ref. [25] is set at $n^{0.6}$.

When the preprocessing of the dataset is completed, 12 feature parameters are selected according to the working conditions of the on-board battery of electric vehicles, i.e., vehicle speed, accumulated mileage, total battery pack current, SOC, insulation resistance, accelerator pedal travel, motor speed, motor temperature, motor controller input voltage, motor controller DC bus current, single unit maximum temperature, single unit median voltage. The calculation results are shown in Table 3. 

The mean value of MIC calculation results of each feature was 0.210 and the features with MIC values greater than the mean were screened out to obtain the maximum information coefficient values of four features greater than the mean value. These four features are cumulative mileage, total battery current, SOC, and median voltage of a single cell. The MIC values of other features are relatively small, indicating that they render little correlation with the total battery voltage.

Moreover, MIC values of the obtained features are normalized to the interval [0, 1], and the normalization results are shown in Table 4. The sorting of all features is obtained by arranging all MIC values in descending order, and it can be observed from Table 4 that the values of accumulated mileage, total battery pack current, SOC, and single unit median voltage are relatively large. Therefore, these four feature parameters and total battery pack voltage, a total of five feature parameters, are selected as the main research objects for analysis in the subsequent sections of this paper. Among the five characteristic parameters, total battery voltage, total battery current, SOC, and median voltage of a single cell are all characteristic parameters of the battery string [26]. Although the accumulated mileage is a characteristic of driving behavior, the accumulated mileage can reflect the accumulated working time of the battery pack from the side, and the battery performance will deteriorate after long-term working [27]. Therefore, the accumulated mileage also contains information affecting the health status of the battery.

## 3. Evaluation Models and Principles

After obtaining the multi-feature parameters of the vehicle battery, the fusion algorithm of multi-feature parameters was studied, and the Gaussian mixture model and Bayesian Inference Distance (GMM–BID) fusion index were proposed to evaluate the health status of the battery.

### 3.1. Feature Fusion Method Based on Gaussian Mixture Model

The Gaussian mixture model (GMM) is based on multiple Gaussian probability density functions (normal distribution curves), which weigh the described object by several Gaussian probability density functions to accurately quantify different objects. Gaussian mixture models are widely used for data classification, such as clustering and image segmentation [28]. From a modeling perspective, the data within an ensemble can be a mixture of a series of individual Gaussian-distributed data. The mathematical formula for the Gaussian mixture distribution can be expressed as follows:

(7) $$p(x)=\sum_{m=1}^{M}\pi_{m}p(\left.x\right|\theta_{m})$$

where $x=\left(x_{1},x_{2,\ldots },x_{d}\right)$ represents a set of data, M represents the number of single Gaussian models in a Gaussian mixture model, $\pi_{m}$ refers to the weight coefficient of a single Gaussian model and the sum of weight coefficients $\pi_{m}$ is 1. $p\left(x|\theta_{m}\right)$ represents the *m*th single Gaussian model with mean value $\mu_{m}$ and the covariance matrix $S_{m}$, i.e.,

(8) $$p(\left.x\right|\theta_{m})=1/{\left(2\pi \right)}^{\frac{d}{2}}S_{m}{}^{\frac{1}{2}}\times exp(-\frac{1}{2}{\left(x-\mu_{m}\right)}^{T}S_{m}^{\left(-1\right)}(x-\mu_{m}))$$

If $\phi =\left\{\pi_{1},\ldots ,\pi_{m};\mu_{1},\ldots ,\mu_{m};S_{1},\ldots ,S_{m}\right\}$, the model can be rewritten as follows:

(9) $$p(x|\phi )=\sum_{m=1}^{M}\pi_{m}p(\left.x\right|\theta_{m})$$

### 3.2. Feature Fusion Indicators

After the above modeling, the GMM model of normal state can be built with fault-free data. Hence, quantitative Bayesian inference-based distance (BID) [29] is used as a quantitative fusion metric to assess the onboard battery health status.

Suppose there are K Gaussian components, where the kth component is $C_{k}$, andthe probability of the occurrence of $C_{k}$ is denoted as $\alpha_{k}$. For the test point $x_{t}$, the probability of belonging to $C_{k}$ can be denoted as $p\left(C_{k}|x_{t}\right)$:

(10) $$p(\left.c_{k}\right|x_{t})=\frac{\alpha_{k}p(\left.x_{t}\right|c_{k})}{p(x_{t})}=\frac{\alpha_{k}p(\left.x_{t}\right|c_{k})}{\sum_{i=1}^{K}\alpha_{k}p(\left.x_{t}\right|c_{k})}$$

where $\alpha_{k}$ can be derived from the modeling data and exhibit a priori probability, as follows:

(11) $$p\left(x_{t}|C_{k}\right)=\frac{1}{{\left(2\pi \right)}^{\frac{1}{2}}|S_{k}{|}^{\frac{1}{2}}}exp\left[-\frac{1}{2}{\left(x_{t}-\mu_{k}\right)}^{T}S_{k}^{-1}\left(x_{t}-\mu_{k}\right)\right]$$

where the mean value of kth Gaussian component is $\mu_{k}$ and the covariance matrix is $S_{k}$. Then, the distance of $x_{t}$ to each component $C_{k}$ can be defined as:

(12) $$D_{\left(C_{k}\right)}(x_{t})={\left(x_{t}-\mu_{k}\right)}^{T}S_{k}^{\left(-1\right)}(x_{t}-\mu_{k})$$

The distance of each component of the test point $x_{t}$ is weighted and summed to find the BID indicator, as follows:

(13) $$\mathrm{BID}=\sum_{K+1}^{K}p(C_{k}|x_{t})D_{\left(C_{k}\right)}(x_{t})$$

**Figure 4:**
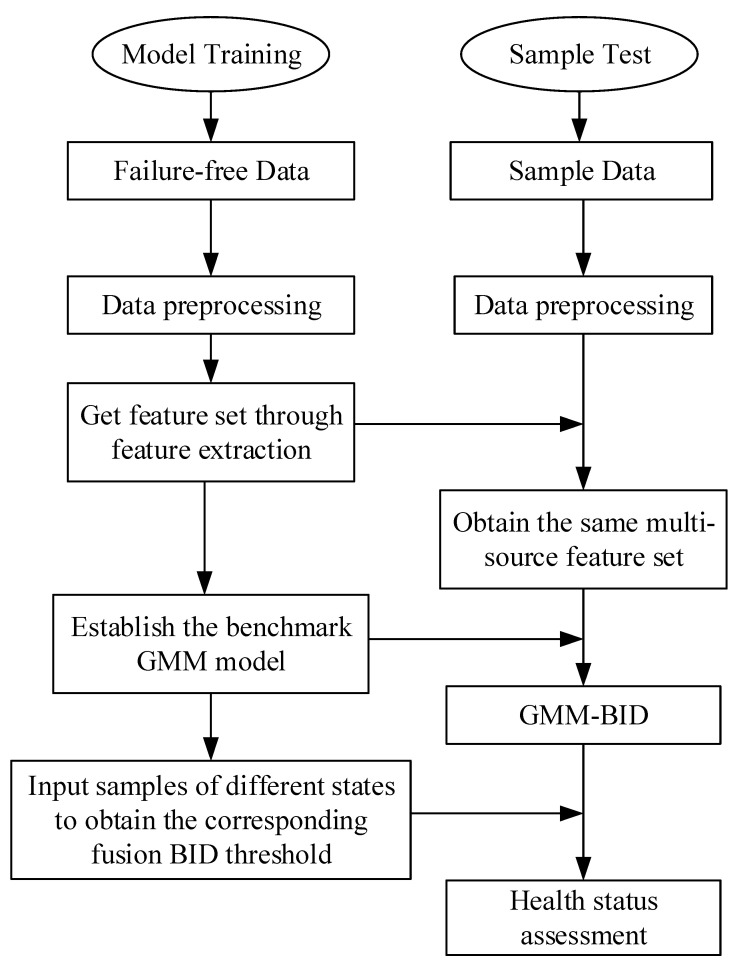
Assessment process based on multi-source information fusion.

The health status evaluation of vehicle batteries based on the multi-source information fusion proposed is presented in Figure 4, showing two key parts: training and testing. The overall evaluation process details are as follows:

The model training process can be given as follows:

- Data preprocessing: First, the fault-free data were preliminarily eliminated, and the numerical data were normalized. Then, the missing data and abnormal data were processed using the weighted moving average and box-type graph method.
- Feature extraction: The total voltage of the battery pack is selected as the feature that can best characterize the state of a battery and the maximum information coefficient is used to extract four features, from among 12 features, with a higher correlation with total voltage.
- The health state GMM model was established using fault-free five-dimensional feature data, and model parameters were determined.
- The three-level fault, two-level fault, one-level fault, and fault-free data samples were input into the health state GMM model to obtain the BID value. The 3-sigma rule was used to establish the BID threshold of different health states.
- The testing process can be summarized as follows:
- The data preprocessing step is the same as the model training stage.
- Feature extraction: According to the features obtained during the model training stage, the feature dataset is extracted.
- The test samples were used as input into the health benchmark GMM model, and the corresponding BID value was calculated.
- The faulty data were judged according to the BID threshold of fault-free data obtained during the model training stage. If the dataset is fault-free, the result could be obtained directly.
- Abnormal data and faulty data judgment: If the BID value falls within the fault threshold range, according to the abnormality/fault judgment rule, and if the consecutive time of BID value within the fault threshold range exceeds 60 s, the dataset is judged as faulty, and the fault type is defined. Otherwise, the dataset is judged as abnormal.

### 3.3. Description of Abnormal Data Judgment Rules

**Figure 5:**
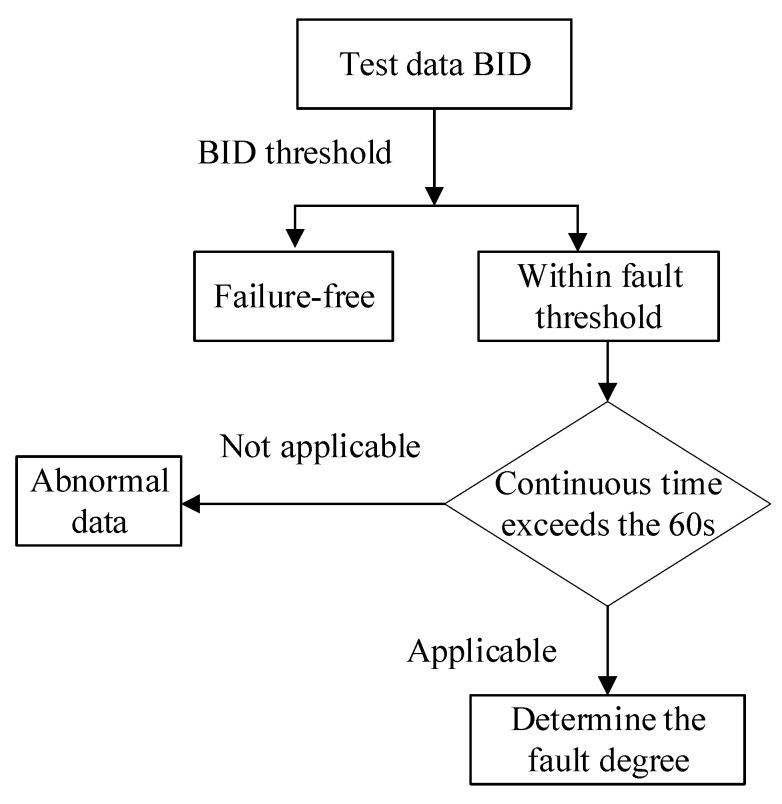
Flowchart of the discriminant rule.

In the health status evaluation of test samples, there may be a problem with abnormal input data. When abnormal data and fault data are input into the GMM model, the BID data may be judged as the result of failure, resulting in inaccurate evaluation results and easy to trigger “false alarm”. To solve the problem that abnormal and faulty data are difficult to distinguish, this paper adds abnormal data determination rules in the process of fault evaluation. The flow chart of the determination rules is shown in Figure 5. Herein, through communication with engineers, based on engineering experience, it is defined that, when the continuous time of BID within the fault threshold exceeds 60 s, the data group is judged as fault data and the degree of fault is determined according to the range of the fault threshold. Otherwise, the data group is judged as abnormal data. If it is set as 30 s or less, abnormal data will be judged as fault data; if it is set as 90 s or more, part of fault data will be judged as abnormal data. According to engineering experience, the determination time is set as 60 s, which can accurately distinguish fault data from abnormal data.

**Figure 6:**
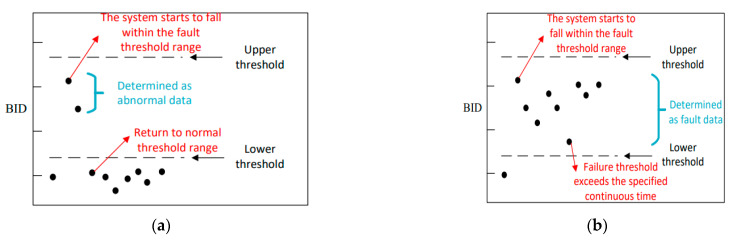
Schematic diagram of the discriminant rule: (**a**) abnormal data determination and (**b**) faulty data determination.

Figure 6 presents a schematic diagram of discrimination, and the discrimination rules are further explained. As shown in Figure 6a, since the second data point, the BID values of two data sets fall into the fault threshold range; however, after two data points, the BID values returned to the normal threshold range. Therefore, the current paper considers that this section of data may be abnormal data; hence, it is not judged as faulty data. However, as shown in Figure 6b, since the second data point, there are more than six data points. Hence, the BID value with a continuous time exceeding 60 s falls within the fault threshold range. Therefore, this dataset is considered as faulty data and the fault degree can be determined by the threshold range.

## 4. Results and Discussion

### 4.1. Model Training

**Figure 7:**
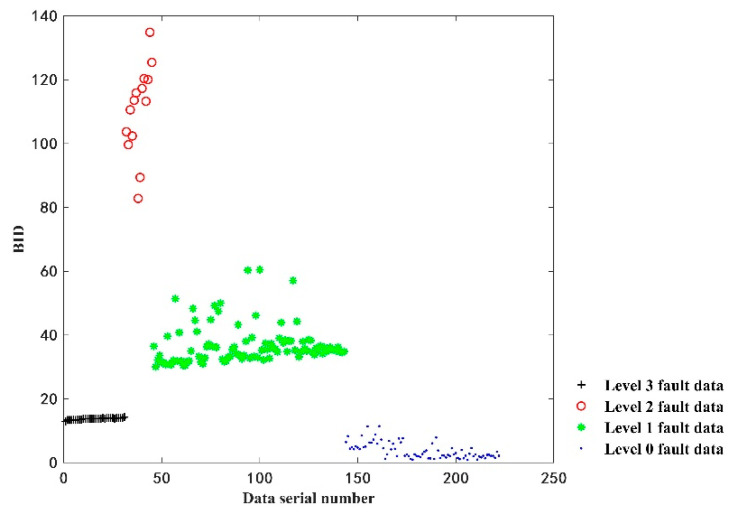
BID fusion results of the vehicle battery.

In order to verify the effectiveness of this model, 222 sets of data are input to the GMM model to obtain the BID value to evaluate the health status of the vehicle battery. Moreover, 222 sets of data are composed of 31 sets of level three faulty data, 14 sets of level two faulty data, 98 sets of level one faulty data and 79 sets of fault-free data. The BID values of the battery pack status data are shown in Figure 7.

As shown in Figure 7, the BID values for the fault-free data are smallest in the range of 0.89 to 11.45. Then, the BID values of the level three fault data ranged from 12.92 to 14.3 and the BID values of the level one fault data ranged from 30.07 to 60.47. Lastly, the BID values of the level two fault data are largest in the range of 82.80 to 134.86. It can also be seen that, except for individual data, such as the 100th group of level one fault data, the other four types of data exhibit fluctuations and are independent of each other.

The BID values obtained for different fault states are used to establish appropriate thresholds and evaluate the degree of battery pack failure. In the actual battery pack operations, the BID value may render fluctuations due to external disturbances. Therefore, this paper adopts a threshold line based on 3σ, i.e., v±3σ, with a confidence level of 99.7% as a criterion to evaluate the health status of a battery pack, where v represents the mean value of each type of data and σ denotes the sample difference of each type of data. Based on 222 groups of data used to establish the threshold line, the results are shown in Table 5. The onboard battery data are input into the GMM model to obtain the BID value and the health status is evaluated based on the threshold range to assess battery failure, as well as estimate the degree of failure.

### 4.2. Health Status Assessment

**Figure 8:**
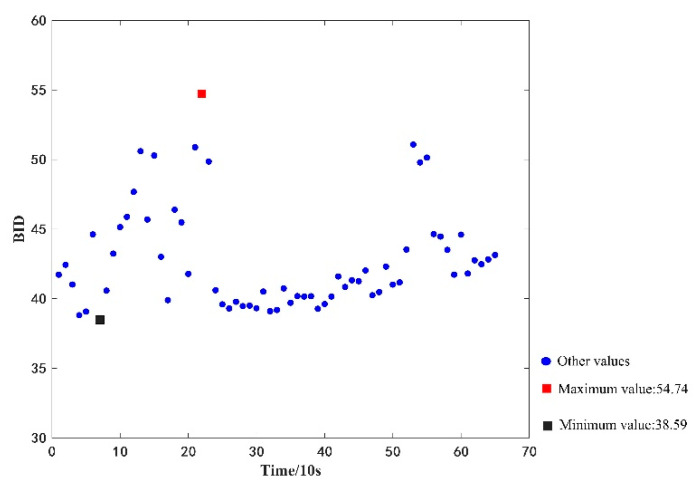
BID values of the faulty dataset.

Herein, the proposed model is first tested by using a total of 65 consecutive sporadic level one fault data of vehicle *LKLA6D1B4KA747050* in the period of 16:10–16:20:40 on 9 June 2020. First, the 65 datasets are pre-processed to standardize the data to a numerical format. Then, from the extracted data types, five-dimensional features of cumulative mileage, total battery pack voltage, total battery pack current, SOC, and single median voltage were retained for calculating the BID value between the GMM model and health state; the maximum value of BID was calculated to be 54.74, whereas the minimum BID value was found to be 38.59. The calculation results are shown in Figure 8.

**Figure 9:**
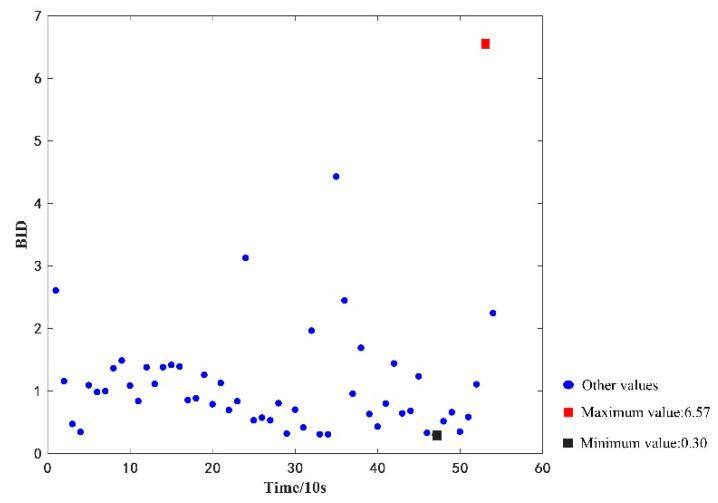
Bid values of the fault-free dataset.

Meanwhile, a total of 54 consecutive time-sensitive fault-free datasets of vehicle *LKLA6D1B6KA749057* in the period of 8:52:29–9:01:19 on 8 September 2020, were tested against the proposed method. Similarly, after data pre-processing, the filtered five-dimensional features were used to calculate the BID values between the test data and health state GMM model, and the maximum and minimum values of BID were calculated to be 6.57 and 0.30, respectively. The calculation results are shown in Figure 9.

Two datasets are compared and analyzed, and it is found that the BID values of both datasets can be separated, indicating that the proposed model can effectively evaluate the health status of a battery pack. Additionally, BID values can distinguish faulty and fault-free states.

### 4.3. Fault State Identification

**Figure 10:**
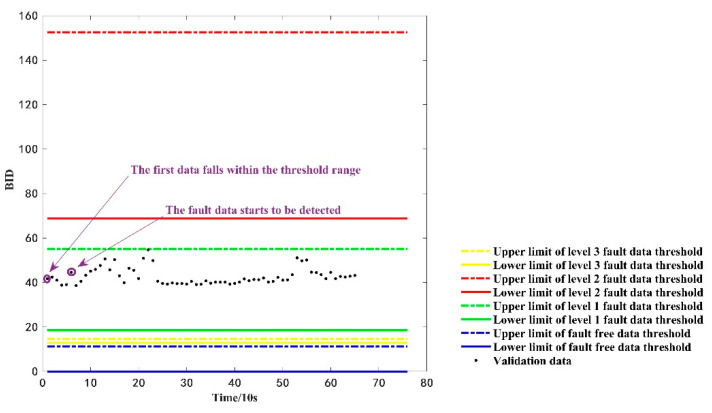
The discrimination results of the verified failure data.

Furthermore, the current paper utilized real vehicle operation data to verify the discrimination rule. First, 65 consecutive time-sensitive first-level fault data in Figure 8 are used for the computational analysis, and their BID values and threshold range of fault types are shown in Figure 10. At the initial momentary point, i.e., momentary point 1 in Figure 10, the BID fusion results fall within the upper and lower threshold range of the first-level fault data. However, the results are retained for evaluation in order to confirm whether the data are abnormal or not until the sixth momentary point, i.e., momentary point 6 in Figure 10. Moreover, the BID fusion results last for 60 s. A first-order fault only occurs when the BID value falls within the upper and lower thresholds of the first-order fault data. Herein, the BID values of more than six consecutive points fall within the upper and lower thresholds of the level one fault data. Hence, the battery is considered to be in level one fault state according to the proposed method, which is consistent with the actual fault type of the data.

**Figure 11:**
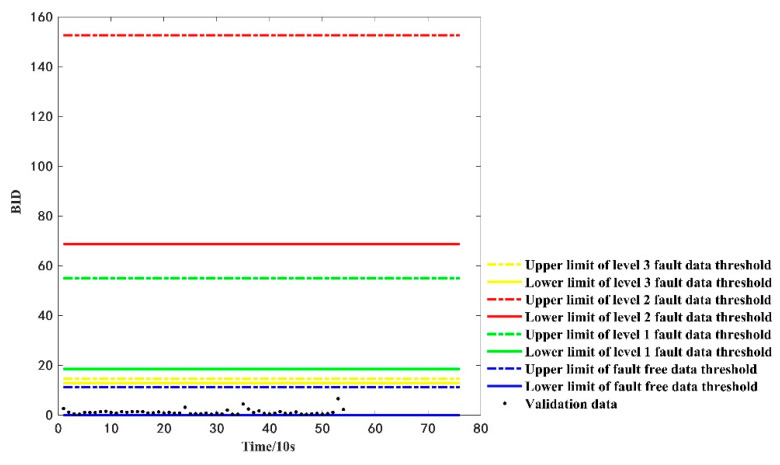
The discrimination results of the verified faults-free data.

Similarly, for the computational analysis using 54 consecutive time-sensitive fault-free data in Figure 9, the BID values and threshold range of the fault type are shown in Figure 11. Figure 11 shows that the BID fusion results always fall within the upper and lower threshold range of the fault-free data, implying that the battery is considered in a fault-free state, which is consistent with the actual fault type of the dataset.

**Figure 12:**
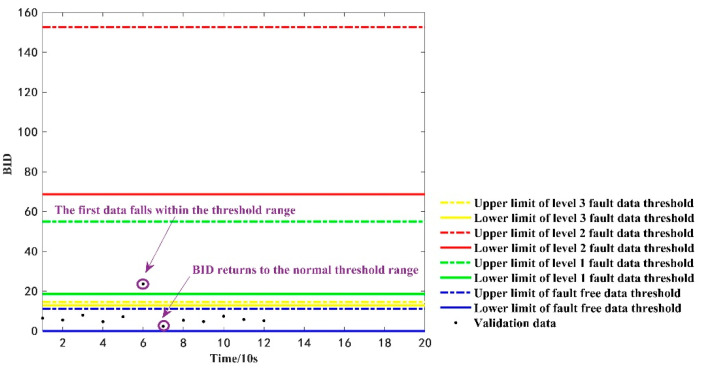
The discrimination results of the verified abnormal data.

To further understand whether a dataset is abnormal or not, the proposed method was tested using 12 consecutive time-sensitive fault-free datasets of the vehicle LKLA6D1B0KA750088 on 30 September 2020, for the period of 10:48:53–10:50:43. The threshold range for calculating its BID value and the fault type are shown in Figure 12. At the sixth momentary point, i.e., momentary point 6 in Figure 8, the BID fusion results fall within the upper and lower thresholds of the first-level fault data. However, in order to confirm whether the dataset is abnormal or not, the evaluation results are retained. At the seventh momentary point, the BID value falls back to the threshold range of normal fault-free data and does not reach the requirement that the BID values always fall within the range for 60 s. The requirements of fault threshold range are not met; hence, this paper does not consider the occurrence of a first-order fault at the sixth moment. Instead, the data at the sixth moment point may be abnormal, which leads to a deviation in the judgment result. The judgment result matches the true fault type of the data and confirms the validity of the proposed judgment rule.

### 4.4. Comparative Analysis with Other Assessment Methods

The GMM–BID method adopted in this paper can effectively distinguish fault states from non-fault states, accurately classify different fault levels, and accurately evaluate the health status of battery packs. In order to demonstrate the advantages of the GMM–BID fusion method adopted in this paper, indicators described based on support vector data description (SVDD) were used to evaluate the battery health status in this paper and were compared with the evaluation results of GMM–BID.

**Figure 13:**
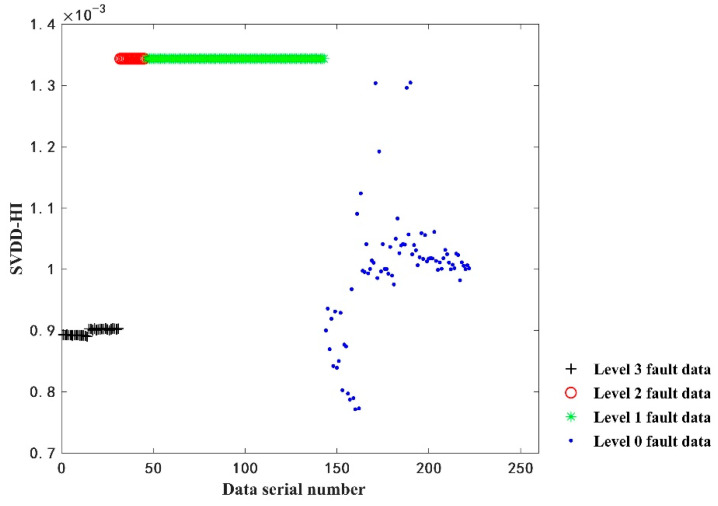
The results of SVDD-HI fusion.

SVDD can solve the small sample classification problem, which at present has been widely used in the study of multi-source information fusion [30,31]. The core idea of SVDD is to obtain a hypersphere with minimum volume by training the same type of data, so that the training data can fall on the hypersphere. For the test data, whether it falls on the hypersphere is calculated; if the test data falls on the hypersphere, it is not such kind of data. In this section, the value of the nuclear distance between the test data and the trained hypersphere is used as a health index (HI) [32]. The SVDD method is used to perform fusion index processing on the 222 sets of data identical to Section 4.1, and the results are shown in Figure 13.

Comparing Figure 13 with Figure 7, it can be found that the evaluation results of SVDD-HI can only distinguish the level three fault data from the other two kinds of fault data to a certain extent, The HI of level one fault data and level two fault data are at the same level, no threshold can be established to distinguish them and the non-fault data and fault data cannot be distinguished. This also illustrates the advantages of using the GMM–BID fusion metric to evaluate battery health.

## 5. Conclusions

In summary, the current work presented an evaluation method to assess the health status of an onboard battery for pure electric vehicles. The following conclusions can be drawn from the current results:

1. The current study provides a solution to the complex multi-dimensional data preprocessing of pure EVs operation. The deletion and weighted sliding averages can be used for processing long-term and short-term missing data, respectively, and the box-type graph method can be used for processing abnormal data. A model based on the preprocessed input data can improve the accuracy of battery evaluation.
2. The maximum information coefficient (MIC), which renders low complexity and high robustness, is selected as the feature selection method. By taking the total battery pack voltage as the benchmark feature, MIC filters the features with a high correlation degree with the total battery pack voltage. Finally, four features, i.e., accumulated mileage, total battery pack current, SOC, and single unit median voltage, are selected to form a featured dataset together with the total battery pack voltage to characterize the on-board battery health status with multi-source information comprehensively.
3. The multi-parameter fusion assessment model of GMM–BID can be fused into a single quantifiable fusion index based on the obtained multi-source data related to battery string status, which can be integrated with various pieces of information to assess the health status of a battery pack, providing useful information for decision making in engineering applications.
4. Herein, the judgment rule is formulated for the battery whose health state is judged as a fault. If the fusion index falls within the fault threshold for more than 60 s continuously, the dataset is judged as a faulty state and the fault type is obtained; otherwise, the dataset is judged as an abnormal data group.

## Figures and Tables

**Table 1:** The data format of a data piece.

Data Type	Data	Data Type	Data
Data Collection Time	20 August 2022 16:43:00	Motor Temperature (°C)	Motor 1:26
Vehicle Status	Start-up	Motor Controller Input Voltage (V)	Motor 1:564.9
Operation Mode	Pure Electric	Motor Controller DC Bus Current (A)	Motor 1:21.0
Speed (km/h)	37.1	Maximum Alarm Level	No fault
Accumulated mileage (km)	447.1	Number of Battery Pack Voltage Subsystem	1
SOC (%)	95	Total Battery Pack Voltage (V)	Device 1:556.1
Gear	Automatic D with Drive No Braking Force	Total Battery Pack Current (A)	Device 1:31.2
Insulation Resistance (kΩ)	59,999	Number of Battery Cells Unit	Device 1:336
Accelerator Pedal Stroke (%)	8	Cell Number(0–335) (V)	(Total 336 cell voltage values)
Number of Motors	1	Number of Battery Temperature Probes Device	Device 1:64
Motor Speed (r/min)	Motor 1:1274	Probe Number (0–335) (°C)	(Total of 64 probe temperature values)

**Table 2:** Short-term missing data interpolation.

**Speed (km/h)**	**Accumulated Mileage (km)**	**SOC (%)**	**Insulation Resistance (kΩ)**	**Motor Speed (r/min)**
7.1	873.8	64	17,503	246
21.3	873.8	64	17,503	730
18.5	874	64	16,206	634
30.5	874.1	64	13,196	1048
0	874.2	64	18,337	0
**Motor Temperature (℃)**	**Motor Controller Input Voltage (V)**	**Motor Controller DC Bus Current (A)**	**Total Battery Pack Voltage (V)**	**Total Battery Pack Current (A)**
51	549.9	49	546.4	65.4
52	551.9	25	549	9.2
52	552	16	548.7	12.8
53	552.9	−1	548.8	−5
53	553.9	1	550.2	3.1

**Table 3:** The results of MIC of parameter.

Feature Parameter	MIC	Feature Parameter	MIC
Vehicle speed	0.055	Motor speed	0.046
Accumulated mileage	0.230	Motor temperature	0.055
Total battery pack current	0.256	Motor controller input voltage	0.114
SOC	0.261	Motor controller DC bus current	0.056
Insulation resistance	0.041	Single Unit maximum temperature	0.052
Accelerator pedal travel	0.195	Single Unit Median Voltage	0.369

**Table 4:** The normalized results of MIC of parameter.

Feature Parameter	MIC	Feature Parameter	MIC
Vehicle speed	0.041	Motor speed	0.015
Accumulated mileage	0.575	Motor temperature	0.040
Total battery pack current	0.657	Motor controller input voltage	0.222
SOC	0.672	Motor controller DC bus current	0.046
Insulation resistance	0	single Unit maximum temperature	0.031
Accelerator pedal travel	0.470	Single Unit Median Voltage	1

**Table 5:** BID thresholds of the battery pack.

Fault Type	Lower Limit of the BID Threshold	Upper Limit of the BID Threshold
Level three fault data	12.91	14.59
Level two fault data	68.73	152.59
Level one fault data	18.55	55.04
Fault-free data	0	11.20

## Data Availability

Not applicable.
